# Super adsorption capability from amorphousization of metal oxide nanoparticles for dye removal

**DOI:** 10.1038/srep09028

**Published:** 2015-03-12

**Authors:** L. H. Li, J. Xiao, P. Liu, G. W. Yang

**Affiliations:** 1 State Key Laboratory of Optoelectronic Materials and Technologies, Nanotechnology Research Center, School of Physics & Engineering, Sun Yat-sen University, Guangzhou 510275, Guangdong, P. R. China

## Abstract

Transitional metal oxide nanoparticles as advanced environment and energy materials require very well absorption performance to apply in practice. Although most metal oxides are based on crystalline, high activities can also be achieved with amorphous phases. Here, we reported the adsorption behavior and mechanism of methyl blue (MB) on the amorphous transitional metal oxide (Fe, Co and Ni oxides) nanoparticles, and we demonstrated that the amorphousization of transitional metal oxide (Fe, Co and Ni oxides) nanoparticles driven by a novel process involving laser irradiation in liquid can create a super adsorption capability for MB, and the maximum adsorption capacity of the fabricated NiO amorphous nanostructure reaches up to 10584.6 mgg^−1^, the largest value reported to date for all MB adsorbents. The proof-of-principle investigation of NiO amorphous nanophase demonstrated the broad applicability of this methodology for obtaining new super dyes adsorbents.

Transitional metal oxide nanoparticles have been studied extensively due to their excellent performances as advanced nanomaterials in areas of environment and energy, and adsorption plays a crucial role in these applications, e.g., strong adsorbing can greatly improve performance^1^,^2^,^3^,^4^,^5^,^6^,^7^,^8^,^9^. Therefore, enhancing adsorption of metal oxide nanoparticles has been a subject that has fascinated scientists and engineers for several decades. Wastewater from textile, pharmaceutical and food processing industry have always been a serious environmental problem, which are harmful to aquatic life and even endanger human health. Many dyes as organic contaminants are usually stable and hardly degradated in conventional wastewater treatment techniques including flocculation, coagulation, chemical precipitation and biological oxidation^10^,^11^. Developing environment friendly adsorption techniques is thus becoming an important issue to remove organic contaminants in water via simply adsorbing^12^,^13^. Methyl blue (MB) is one of the most common dyes which is widely used as the coloring agent and disinfector in pesticides, pharmaceuticals, dyestuffs and varnishes^14^,^15^,^16^, and it is generally adopted as a representative organic pollutant to test the adsorption performance for the removal of organic contaminants from wastewater. However, up to date, the maximum adsorption capacity of all the reported MB adsorbents is still below 10^4^ mgg^−1^ (Supplementary Information S1).

Here, we synthesize amorphous transitional metal oxide (Fe, Co and Ni oxides) nanoparticles by a simple and green process, i.e., laser irradiation in liquid. Our measurements indicate that the maximum adsorption capacity of the fabricated amorphous nanostructures of metal oxide nanoparticles for MB is beyond 10^4^ mgg^−1^ and high up to 10584.6 mgg^−1^, which is the largest value reported to date for all MB adsorbents. These findings thus promote the application of amorphous nanostructures as advanced adsorbent materials.

## Results

### Morphology and structural characterizations

**Figure 1:**
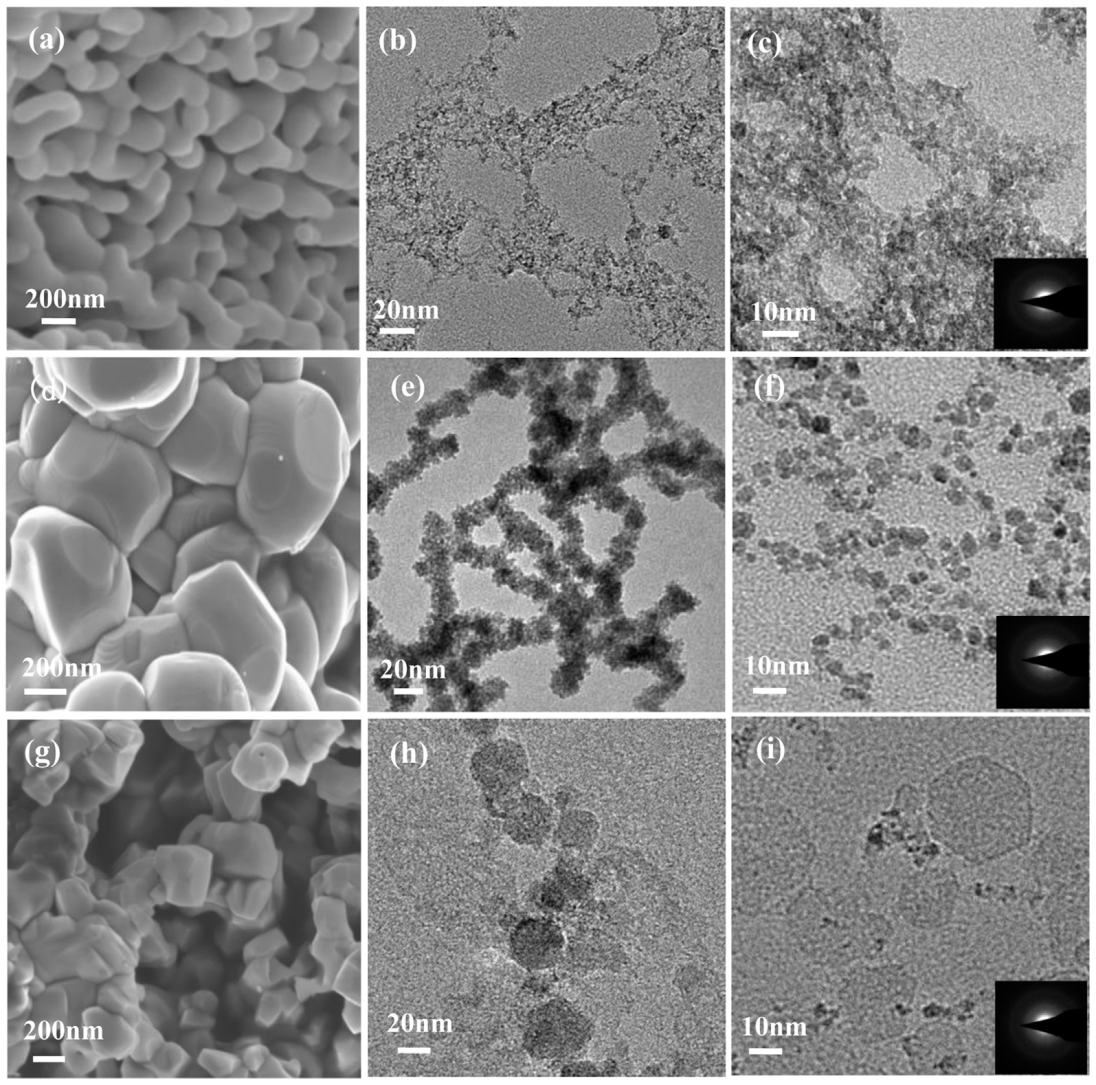
The morphology and structure evolution of the starting metal oxides nanoparticles upon the laser irradiation in liquid. (a), (d) and (g) TEM iamges of the starting nanoparticles of Fe_2_O_3_, CoO and NiO, respectively. (b–c), (e–f) and (h–i) TEM images of the synthesized products of Fe_2_O_3_, CoO and NiO, respectively. The insets in (c), (f) and (i) are the corresponding SAED patterns of the Fe_2_O_3_, CoO and NiO samples, respectively.

The morphology and structure evolution of the starting metal oxides nanoparticles upon the laser irradiation in liquid is shown in Fig. 1. From these scanning electron microscopy (SEM) and Transmission electron microscopy (TEM) images, we can see the evolution that the particles are becoming smaller. The final products are the amorphous nanostructures as shown in the selected area electron diffraction (SAED) image insets in Fig. 1. Meanwhile, the corresponding X-ray diffraction (XRD) patterns reveal the detailed structural evolution of the starting raw materials from crystal to amorphous (Supplementary Information S2). Therefore, these results show that the amorphization of transitional metal oxides nanoparticles driven by the laser irradiation in liquid is achieved. Note that the Brunauer-Emmett-Teller (BET) surface areas of the synthesized products are promoted to be larger 10–30 times than that of the starting nanoparticles (Supplementary Information S3).

### Effects of pH on adsorption

**Figure 2:**
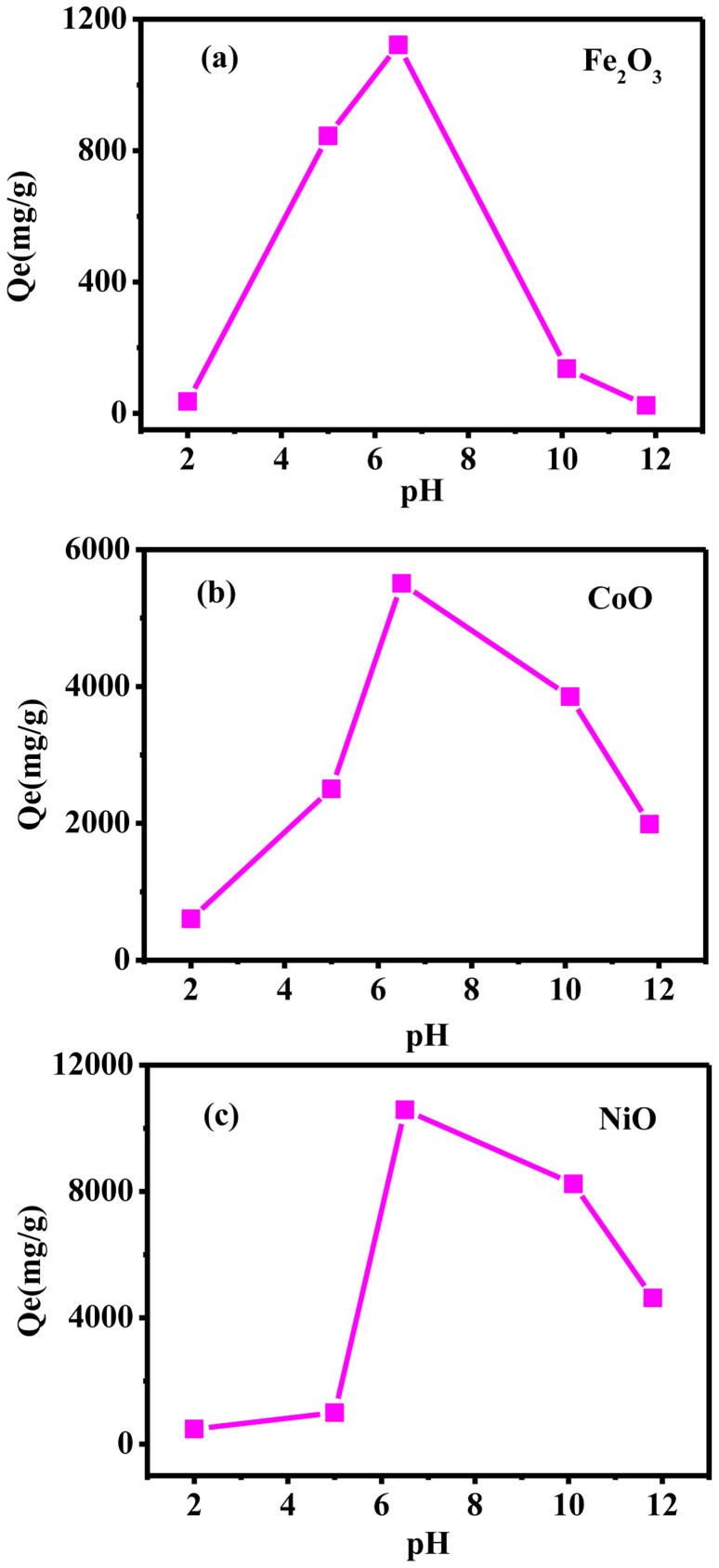
The effect of initial pH on adsorption. (a) Fe_2_O_3_, (b) CoO and (c) NiO.

It is well known that the effect of pH plays a significant role on the active sites of nano-adsorbents as well as the dye species during the adsorption reaction. The experiments are carried out in pH range 2.0–11.8 and the results are illustrated in Fig. 2. The chart clearly reveals that the removal degree of MB increases as the pH value rises from 2 to 6.5, and then decreases rapidly at higher pH value. The optimum pH for the samples is 6.5. At acidic pH, it is obviously that the adsorption capacity of MB decreased at low pH is explained by the fact that the leaching of Fe, Co, Ni, which might destroy the structure of the samples. At alkaline pH, decreasing MB adsorption at high pH may be due to the competition of OH with MB ions for the adsorption sites on the samples. The increasing number of hydroxyl groups decreases the number of positively charged sites and reduces the attraction between dye and adsorbent surface. Therefore, the possible mechanism of MB adsorption is considered as the strong electrostatic interaction between the positive active site of the adsorbent and the negative charge of MB.

### Adsorption isotherm

**Figure 3:**
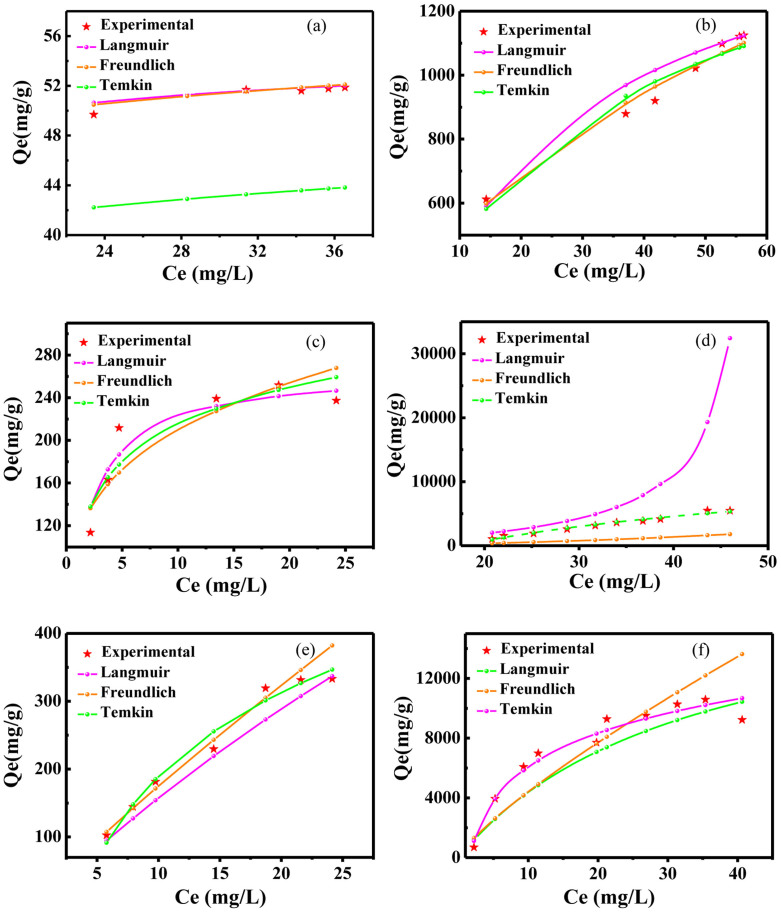
Comparison of the equilibrium isotherms between the experimental and theoretical data. (a–c) The starting nanoparticles of Fe_2_O_3_, CoO and NiO, respectively. (d–f) The fabricated amorphous nanostructures of Fe_2_O_3_, CoO and NiO, respectively.

To comprehend the adsorption potential between adsorbent and adsorbate, the working adsorption isotherm modes such as Langmuir, Freundlich and Temkin need to be determined (Supplementary Information S4). The adsorption experiments are conducted at 298 K and pH = 6.5. The adsorption isotherms are illustrated in Fig. 3. From these data, we can see that, the CoO and NiO samples work in the Temkin mode, and the Fe_2_O_3_ sample works in the Freundlich mode. Meanwhile, we can see that the adsorption capacity of the fabricated colloidal solution is promoted to be higher 20 to 30 times than that of the starting raw materials. Importantly, the maximum MB adsorption capacity of the synthesized NiO colloidal solution is beyond absorption limit of 10^4^ mgg^−1^ and high up to 10584.6 mgg^−1^, which is the largest value reported to date for all MB adsorbents.

### Adsorption kinetics

**Figure 4:**
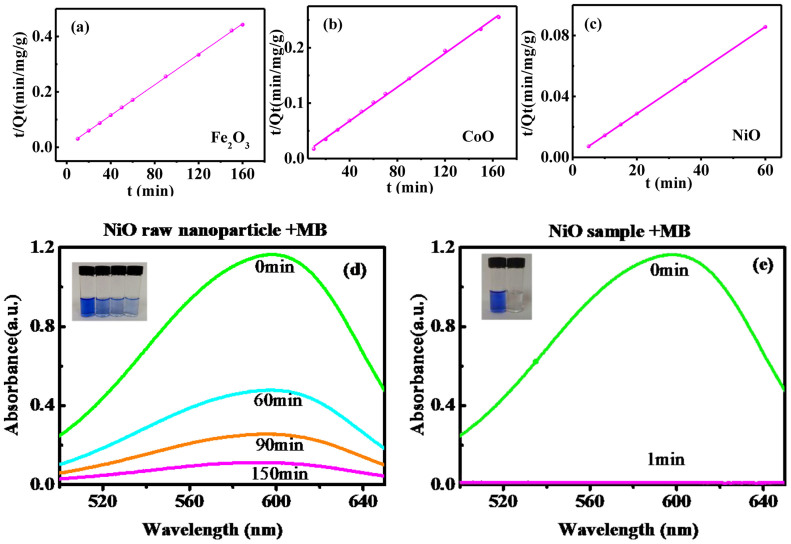
Adsorption kinetic results. (a–c) All three samples exhibt the pseudo second-order kinetic behaviors. The adsorbing rates of the starting NiO nanoparticles (d) and the synthesized amorphous nanostructures (e).

The adsorption kinetics of MB onto amorphous nanostructures of metal oxides nanoparticles are evaluated by utilizing the pseudo first-order and pseudo second-order models (Supplementary Information S5). These results (Fig. 4a–c) show that the pseudo-second-order kinetic model fits well the adsorption kinetics of MB onto the samples in our case, and the rate limiting step would be chemisorptions^17^. Clearly, we can see that MB can be removed 99.0% via the NiO sample in 1 min., while 90.3% via the starting raw nanoparticles in 150 min.

## Discussion

### Mechanism of MB adsorption onto amorphous transitional metal oxides

The super adsorption capacity induced by the amorphization of transitional metal oxides nanoparticles is attributed to these reasons below. Firstly, from Supplementary Information S3, we can clearly see that the BET surface areas of the synthesized products are larger 10–30 times than that of the starting nanoparticles. Thus, larger surface area created by the amorphization of transitional metal oxide nanoparticles is very beneficial to the adsorption performance of the synthesized products.

**Figure 5:**
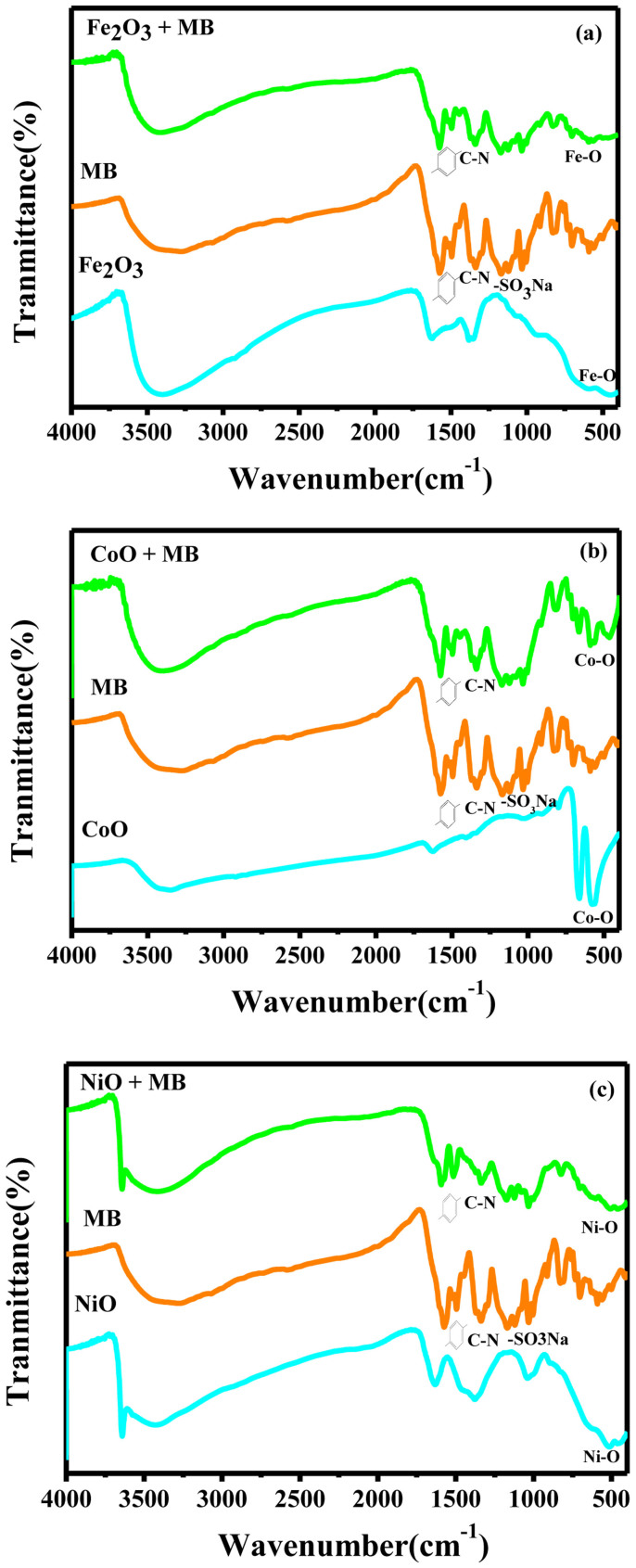
FTIR spectra of the fabicated colloidal solutions, the MB solution and the colloidal solutions adsorbed by MB. (a) Fe_2_O_3_, (b) CoO and (c) NiO.

Secondly, we systemically compare the surface states of the starting raw materials and the synthesized colloidal solutions, and that of the synthesized materials before and after adsorbing MB by Fourier Transform Infrared Spectroscopy (FTIR) as shown in Fig. 5 and Supplementary Information S6. Clearly, we can see that the FTIR spectra of the starting raw materials has not basically the adsorption peaks, but a lot of OH radicals have been generated in the synthesized colloidal solutions. After adsorbing MB, the aromatic ring vibrations at 1575, 1495 and 1449 cm^−1^, the C-N stretching vibration at 1337 cm^−1^ and the SO_3_Na radical vibrations at 1169, 1121, 1032 and 1005 cm^−1^ emerge in the spectra of the fabricated colloidal solutions, which reflecting the evidence that chemical bonds are very strong in this case. Since the reaction ability of OH radicals is high enough to attack almost any organic molecules such as SO_3_Na radicals, it is assigned as a key species in the adsorption mechanism of many hazardous chemical compounds. Therefore, these results reveal the strong interaction between MB and amorphous nanostructures. Note that M (Fe, Co and Ni)-O bonds are the main vibrations in the low wave number. We can infer that between M^2+^ in M (OH^+^) and O^2−^ in sulfonic groups in MB exits strong ionic bonding. The amorphization of transitional metal oxides nanoparticles provides much more active sites as adsorption sites and hangs more function groups which greatly promote the adsorbing performance.

Thirdly, the surface charge of the synthesized colloidal solutions plays a key role in the MB adsorption. Considering the pHpzc of the amorphous transitional metal oxide (Fe, Co and Ni oxides) nanoparticles, (pHpzc = 8.7, 10.5, 10.7 for Fe_2_O_3_, CoO, NiO respectively) the surface formation of M(Fe, Co and Ni)O is mainly M(OH^+^) at pH = 6.5 in our experiments (Supplementary Information S7). Hence, the interaction between MB and M(Fe, Co and Ni)O might be mainly the ionic bonding between the positively charged center of M(Fe, Co and Ni) (OH^+^) and negatively charged functional groups of MB(-SO_3_^−^). Thus, this strong interaction also improves adsorption. Additionally, the zeta potential values (Supplementary Information S7) verify the deduction above.

### Transformation mechanism of crystals nanoparticles into amorphous ones

The reasons that the transformation from crystals nanoparticles into amorphous ones under laser irradiation in liquid can be ascribed to two aspects below. For one thing, the laser energy with larger power density (400 mJ/pulse in our case) is sufficient to destroy the order crystalline structure. Compared with crystal, amorphous usually keeps the larger cohesive energy, which means that they are unstable state that can be synthesized in the nonequilibrium condition created by laser ablation in liquid. For another, water environment may be essential for the formation of amorphous. Khan *et. al.* claimed that they used laser to ablate Ni plate in water to produce amorphous NiO NPs^14^. Also, Shim and his co-worker used laser to ablate Fe_2_O_3_ target in water and obtain amorphous Fe_2_O_3_. However, they got crystal when they choose alcohol and acetone as liquid^15^. Therefore, these evidences indicate that the water may easily form amorphous layer or benefit for the generation of amorphous for metal oxides.

### Capacity comparison and selectivity of MB adsorption onto amorphous transitional metal oxides

We compare the adsorption performances of the synthesized colloidal solutions for MB, methylene blue (YMB) and methyl orange (MO) (Supplementary Information S8). Definitely, these colloidal solutions show relatively poor performances in the processes of YMB and MO adsorption, which is attributed to the absence of SO_3_Na function groups in YMB and MO. MB, MO and YMB have three, one and zero SO_3_Na, respectively. Therefore, the adsorption of the synthesized colloidal solutions is highly selective.

In summary, a series of the amorphization of transitional metal oxide nanoparticles have been achieved and the related amorphous nanostructures as super adsorption materials have been fabricated by a simple and environmentally friendly laser irradiation in liquid. The synthesized colloidal solution of amorphous NiO performed a super adsorption capacity for MB, especially the maximum MB adsorption capacity is beyond absorption limit of 10^4^ mgg^−1^ and reaches up to 10584.6 mgg^−1^, which is the largest value reported to date for MB adsorbents. MB can be removed 99.0% via amorphous NiO in 1 min, while 90.3% via the starting raw nanoparticles in 150 min. Therefore, these findings demonstrated the broad applicability of this methodology for accessing new super dyes adsorbents.

## Methods

### Materials synthesis

Iron (III) oxide, Nickel (II) oxide, Cobalt (II) oxide, Methyl Orange and Methylene blue are from Alfa Aesar (China), and Methyl blue is from Sigma-Aldrich (China). All chemicals are of analytical grade. All solutions are prepared with deionized water. The laser irradiation in liquid technique has been reported in our previous works^18^,^19^,^20^,^21^,^22^,^23^,^24^,^25^,^26^. In this case, 5 mg metal oxide nanoparticles powder with a purity of 99.99% is firstly placed in a glass bottle filled with 10 ml of deionized water. Magnetically-stirred operation is to make the powder monodisperse in water. Then, the aqueous solution is irradiated by a Q-switched Nd:YAG laser device with a wavelength of 532 nm, repeating frequency of 10 Hz, pulse width of 10 ns, energy density of 400 mJ/pulse and spot size of 4 mm in diameter. During laser irradiating, the liquid environment is maintained at ambient temperature and pressure. The laser irradiation process lasts for 30 min. Then, the colloidal solution is synthesized and then collected in a cuvette for measurements.

### Materials characterization

The materials are characterized by SEM with Thermal Field Emission Environmental SemEdsEBSD. XRD is performed with a Rigaku D-max 2200 VPC (Japan) with Cu Kα radiation (λ = 1.54056 Å, accelerating voltage is 40 kV, emission current is 26 mA), and a scanning rate of 1° s^−1^ is employed. TEM is carried out with a JEOL JEM-2010HR instrument (Japan) at an accelerating voltage of 200 kV. The samples are ultrasounded for 30 minutes and then dropped pipette onto a ultrathin carbon supported film. Above mentioned techniques are used to identify morphology, structure and composition of the as-synthesized samples. The optical absorption of the colloidal dispersion is measured by using the UV-Vis-NIR 3150 Spectrophotometer (Japan). The nitrogen adsorption-desorption isotherms are measured by Automated gas sorption analyzer Autosorb-iQ2-MP (USA) at 77 K. And the specific surface area of the materials is calculated by the Brunauer-Emmett-Teller (BET) theory. The Zeta potential is carried out by Nanoparticle size-Zeta potential and molecular weight analyzer (Brookhaven). The Inductively coupled plasma-atomic emission spectrometry (ICP-AES) using a ThermoFisher Icap6500Duo is employed to analyze the concentration of Fe, Co, Ni, with an incident power of 1150 W, a plasma gas flow of 14 L/min, and an atomization gas flow of 0.6 L/min. FTIR of the samples are recorded with the Fourier transformation infra-red spectrometer coupled with infra-red microscope (EQUINOX 55).

### Effects of pH on adsorption

In order to evaluate the influence of pH on the adsorption capacity of the samples, experiments are carried out at initial concentration of 60 mg/L (pH > 7) and 6 mg/L (pH < 7) and in the range of 2.0–11.8, the equilibrium time of 12 h at room temperature. The initial pH of the solutions is adjusted with 0.5 mol/L HCl and 0.5 mol/L NaOH solutions.

### Adsorption kinetic experiments

All adsorption kinetic experiments are performed at ambient temperature and pressure. The adsorption isotherm is studied by adding 1 ml of 415 mg/L fresh fabricated colloid solution (1 ml of 350 mg/L for Fe_2_O_3_ and CoO colloid solution) to different volume (5–200 ml) of 60 mg/L MB aqueous solution. Then, the solution is continuously stirred in dark at a constant speed of 150 rpm for 12 h to ensure the established of an adsorption-desorption equilibrium among the sample, MB, and water. Then, aliquots (5.0 ml) of the samples are collected, centrifuged (centrifugation speed is 13500 rpm for 10 minutes), filtered (Millipore, 0.22 μm). The kinetics (time ranging from 0–60 min for the NiO sample, time ranging from 0–165 min for the Fe_2_O_3_ and CoO sample) is studied by adding 30 ml of 415 mg/L fresh fabricated NiO colloid solution (350 mg/L for Fe_2_O_3_ and CoO colloid solution) to 150 ml 60 mg/L MB aqueous solution. Then, morsels (5.0 ml) of samples are collected, centrifuged immediately to separate the suspended solid and filtered (Millipore, 0.22 μm) for further research at suitable time intervals. The determination are carried out by logging the maximum absorption using a UV-vis spectrometer at λ_max_ = 595 nm (pH = 6.5). All experiments are performed in duplicate. In order to judge the adsorption performance of the as-synthesized samples, the raw powders (the starting nanoparticles) and commercial activated carbon are introduced for comparison. The concentration reserved in the adsorbent phase (*Q_e_*, mg/g) is measured by using the following equation
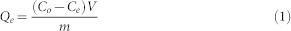
where *C_e_*(mg/L) is the initial dye concentration, *C_t_*(mg/L) is the equilibrium dye concentration in the aqueous solution, *V*(L) is the volume of solution and *m*(g) is the mass of the adsorbent. The adsorption capacity of the A-MONP at time *t*, *Q_t_*(mg/g) was also calculated
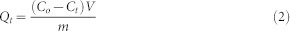
where *C_t_* is the concentration of MB at time *t*(mg/L).

## Author Contributions

G.W.Y. designed the experiments; L.H.L. and J.X. carried out the experiments; P.L. and J.X. carried out data analysis; L.H.L. and G.W.Y. wrote the paper.

## Supplementary Material

Supplementary Information Super adsorption capability from amorphousization of metal oxide nanoparticles for dye removal
