# Supplementary material for: Super adsorption capability from amorphousization of metal oxide nanoparticles for dye removal
Source: Sci Rep. 2015 Mar 12;5:9028. doi: 10.1038/srep09028 (PMC4356953; doi:10.1038/srep09028)
Supplement: Supplementary Information — Super adsorption capability from amorphousization of metal oxide nanoparticles for dye removal [file srep09028-s1.doc]

**Supplementary Information for**

**Super adsorption capability from amorphousization of metal oxide nanoparticles for dye removal**

L. H. Li, J. Xiao, P. Liu, G. W. Yang

*State Key Laboratory of Optoelectronic Materials and Technologies, Institute of Optoelectronic and Functional Composite Materials, Nanotechnology Research Center, School of Physics & Engineering, Sun Yat-sen University, Guangzhou 510275, Guangdong, P. R. China*

**S1 Comparison of adsorption results for removing MB with other adsorbents in previous reports.**

| Adsorbent | Qmax(mg/g) | T(k) | References |
| --- | --- | --- | --- |
| Activated charcoal | 24.79 | 298 | 1 |
| Fe_3_O_4_ | 68.5 | 291 | 2 |
| CoFe_2_O_4_ | 244.5 | 291 | 3 |
| MnFe_2_O_4_ | 497.512 | 298 | 3 |
| Chemically reducedgraphene oxide | 1519.6 | 303 | 4 |
| Magnetic β-cyclodextrin-chitosan nanoparticles | 2783.304 | 303 | 5 |
| ZnO nanoparticles | 7918.02-9197.7 | 303 | 6 |
| **Fe_2_O_3_ sample** | **1124.703** | 298 | **This work** |
| **CoO sample** | **5501.926** | 298 | **This work** |
| **NiO sample** | **10585** | 298 | **This work** |

**References:**

1. Iqbal, M.J. & Ashiq, M.N. Adsorption of dyes from aqueous solutions on activated charcoal. *J. Hazard Mater.* **139**, 57-66 (2007).

2. Wang, L., Li, J., Wang, Y., Zhao, L. & Jiang, Q. Adsorption capability for Congo red on nanocrystalline MFe_2_O_4_ (M=Mn, Fe, Co, Ni) spinel ferrites. *Chem. Eng. J.* **181–182,** 72-79 (2012).

3. Yang, L. et al. The investigation of synergistic and competitive interaction between dye Congo red and methyl blue on magnetic MnFe_2_O_4_. *Chem. Eng. J.* **246**, 88-96 (2014).

4. Wu, T. et al. Adsorption characteristics of acrylonitrile, p-toluenesulfonic acid, 1-naphthalenesulfonic acid and methyl blue on graphene in aqueous solutions. *Chem. Eng. J.* **173**, 144-149 (2011).

5. Fan, L. et al. Synthesis and characterization of magnetic beta-cyclodextrin-chitosan nanoparticles as nano-adsorbents for removal of methyl blue. *Int. J. Biol Macromol***.50**, 444-450 (2012).

6. Zhang, F. et al. Adsorption behavior and mechanism of methyl blue on zinc oxide nanoparticles. *J. Nanopart. Res.* **15,**2034-2043 (2013).

**S2 XRD patterns of the starting raw materials from crystal to amorphous.**

**S3 BET surface areas of raw nanoparticles and samples**

| **Raw nanoparticles** | **BET surface**  **area (m^2^/g)** | **Samples** | **BET surface**  **area (m^2^/g)** | **S_A-MONP_/ S_MONP_** |
| --- | --- | --- | --- | --- |
| Fe_2_O_3_ | 8.529 | Fe_2_O_3_ | 273.504 | **32.067** |
| CoO | 8.494 | CoO | 97.269 | **11.451** |
| NiO | 6.889 | NiO | 182.781 | **26.532** |

**S4 Adsorption isotherm mode**

The Langmuir mode predicts that each active site can only hold one adsorbate molecule and that the adsorption takes place on homogeneous sites within the adsorbent. There is no interacting between the adsorbed species. The linear equation given by the Langmuir is expressed as^1^

(1)

where (mg/g) and (mg/L) are the capacity of adsorbed MB onto samples and MB concentration at equilibrium, respectively. (mg/g) is the maximum capacity of MB adsorbed per unit mass of samples, and $b$(L/mg) is a constant related to the adsorption energy. Plotting versus generates a straight line with the slope and intercept , then and$b$are obtained.

The Freundlich mode is based on the assumption that the adsorption takes place on heterogeneous surface which have different adsorption energies. This model is expressed by^2^

(2)

where is a constant related to adsorption capacity (mg/g) and $1/n$ is an empirical parameter connected to surface heterogeneity or adsorption intensity. It is generally stated that values of $n$ in the rang 2-10 means good, 1-2 moderately difficult, and less than 1 poor adsorption characteristics^1^. Additionally, the Freundlich mode in linear form is

(3)

the Freundlich constants and $1/n$ can be determined from the intercept and slope of linear plot of against l, respectively.

The Temkin mode assumes that the heat of adsorption of all molecules decreases linearly with coverage due to adsorbate interactions. The linear form of Temkin is expressed as^3^

(4)

where B (J/mol) is the constant related to heat of adsorption and A (L/g) is the equilibrium binding constant corresponding to the maximum binding energy.

**Langmuir, Freundlich and Temkin Isotherm Prameters for MB onto Samples**.

| **Samples** | **Langmuir** | | | **Freundlich** | | | **Temkin** | | |
| --- | --- | --- | --- | --- | --- | --- | --- | --- | --- |
|  | **Q_max_ (mg/g)** | **B**  **(L/mg)** | **R^2^** | **K_F_**  **(mg/g(L/mg)^1/n^)** | **N** | **R^2^** | **A**  **(L/mg)** | **B** | **R^2^** |
| Fe_2_O_3_ | 1623.65 | 0.04 | 0.94058 | 183.3762 | 2.2486 | **0.9746** | 0.334 | 371.8988 | 0.9342 |
| CoO | -2839.47 | -0.02 | 0.725 | 3.375185 | 0.5109 | 0.9697 | 0.0572 | 5542.57 | **0.9752** |
| NiO | 18992.27 | 0.03 | 0.46772 | 691.4632 | 1.2426 | 0.8084 | 0.6324 | 3287.519 | **0.9511** |

**Adsorbing Capacities of MB onto Raw nanoparticles and Samples.**

| **Raw nanoparticles** | **Q_max_ (mg/g)** | **Samples** | **Q_max-A_(mg/g)** | **Q_max-A_/Q_max_** |
| --- | --- | --- | --- | --- |
| Fe_2_O_3_ | 54.64 | Fe_2_O_3_ | 1124.73 | **21** |
| CoO | 267.38 | CoO | 5501.93 | **21** |
| NiO | 332 | NiO | 10584.6 | **32** |

**References:**

1. Langmuir, I. The adsorption of gases on plane surfaces of glass, mice and platinum. *J. Am. Chem. Soc.* **40**, 1361-1403 (1918).

2. Duran, C., Ozdes, D., Gundogdu, A. & Senturk, H.B. Kinetics and Isotherm Analysis of Basic Dyes Adsorption onto Almond Shell (Prunus dulcis) as a Low Cost Adsorbent. *J. Chem. Eng. Data* **56**, 2136-2147 (2011).

3. Haghseresht, F. & Lu, G.Q. Adsorption Characteristics of Phenolic Compounds onto Coal-Reject-Derived Adsorbents. *Energy Fuels.* **12**, 1100-1107 (1998).

4. Kalavathy, M.H., Karthikeyan, T., Rajgopal, S. & Miranda, L.R. Kinetic and isotherm studies of Cu(II) adsorption onto H_3_PO_4_-activated rubber wood sawdust. *J. Colloid Interface Sci.* **292**, 354-362 (2005).

**S5 Adsorption kinetics**

The pseudo first-order model is usually applied to the initial stage of an adsorption process. The model is expressed by the following equation^1^

(1)

where and are the amounts of MB adsorbed (mg/g) at equilibrium and at any time, $t$ (min), respectively, and is the equilibrium rate constant of the pseudo first-order sorption (1/min). The equation of pseudo second-order model is illustrated as^2^

(2)

where (g/mg/min) is the equilibrium rate constant of pseudo second-order. (mg/g) is the maximum adsorption capacity and (mg/g) is the adsorbing capacity at time $t$ (min).

**Determined Constants of Pseudo-first-order and Pseudo-second-order kinetic Model of MB adsorption onto Samples for 60 mg/L initial concentration.**

|  | **Pseudo-first kinetics model** | | | **Pseudo-second kinetics model** | | |  |
| --- | --- | --- | --- | --- | --- | --- | --- |
| **Samples** | k_1_  (min^-1^) | Qe  mg/g) | R^2^ | k_2_  (g/mg·min) | Qe  (mg/g) | R^2^ | Qe-exp  (mg/g) |
| Fe_2_O_3_ | 0.00154 | 126.4845 | 0.7896 | 0.001548 | 362.3188 | **0.9998** | 461.402 |
| CoO | 0.00617 | 139.0398 | 0.9402 | 0.000354 | 653.5948 | **0.9984** | 694.3212 |
| NiO | 0.00941 | 18.12768 | 0.962 | 0.012175 | 699.3007 | **0.9999** | 710.9182 |

**Plots of Samples adsorb MB within short time.**

**Pseudo first-order Model and Pseudo second-order Model for Samples.**

**References:**

1. Doğan, M., Alkan, M., Demirbaş, Ö., Özdemir, Y. & Özmetin, C. Adsorption kinetics of maxilon blue GRL onto sepiolite from aqueous solutions. *Chem. Eng. J.* **124**, 89-101 (2006).

2. Wu, C.-H. Adsorption of reactive dye onto carbon nanotubes: Equilibrium, kinetics and thermodynamics. *J. Hazard Mater.* **144**, 93-100 (2007).

**S6 FTIR Spectra of the Raw nanoparticles and Samples.**

**S7** **Zeta potential values of Samples at different pH values (at 298K).** (a) Fe_2_O_3_, (b) CoO and (c) NiO.

**Zeta potential value of Samples.**

| **Samples** | **Z_1_(mv)** | **Z_2_(mv)** | **Z_3_(mv)** | **Average(mv)** |
| --- | --- | --- | --- | --- |
| Fe_2_O_3_ | 34.98 | 29.45 | 32.22 | **32.2167** |
| CoO | 43.14 | 42.97 | 33.29 | **39.8** |
| NiO | 55.98 | 50.43 | 52.87 | **53.0933** |

**S8 Ad**sorptions of the synthesized colloidal solutions for MB, YMB and MO

| **Samples** | **Q_max_ -MB**  **(mg/g)** | **Q_max_ -YMB**  **(mg/g)** | **Q_max_ -MO**  **(mg/g)** |
| --- | --- | --- | --- |
| Fe_2_O_3_ | 1124.73 | 68.32 | 9.73 |
| CoO | 5501.93 | 34.37 | 11.53 |
| NiO | 10584.61 | 34.46 | 129.45 |

**Chemical structure of (a) MB, (b) MO and (c) YMB.**

(a)

(b)

(c)
